# Multivariate analysis of FcR-mediated NK cell functions identifies unique clustering among humans and rhesus macaques

**DOI:** 10.3389/fimmu.2023.1260377

**Published:** 2023-12-06

**Authors:** Marina Tuyishime, Rachel L. Spreng, Brady Hueber, Junsuke Nohara, Derrick Goodman, Cliburn Chan, Richard Barfield, Whitney E. Beck, Shalini Jha, Stephanie Asdell, Kevin Wiehe, Max M. He, David Easterhoff, Haleigh E. Conley, Taylor Hoxie, Thaddeus Gurley, Caroline Jones, Nihar Deb Adhikary, Francois Villinger, Rasmi Thomas, Thomas N. Denny, Michael Anthony Moody, Georgia D. Tomaras, Justin Pollara, R. Keith Reeves, Guido Ferrari

**Affiliations:** ^1^ Department of Surgery, Duke University, Durham, NC, United States; ^2^ Duke Human Vaccine Institute, Durham, NC, United States; ^3^ Center for Human Systems Immunology, Durham, NC, United States; ^4^ Department of Biostatistics and Bioinformatics, Duke University, Durham, NC, United States; ^5^ Department of Medicine, Duke University, Durham, NC, United States; ^6^ New Iberia Research Center, University of Louisiana at Lafayette, New Iberia, LA, United States; ^7^ U.S. Military HIV Research Program, Walter Reed Army Institute of Research, Silver Spring, MD, United States; ^8^ Department of Pediatrics, Duke University, Durham, NC, United States; ^9^ Department of Integrative Immunobiology, Duke University, Durham, NC, United States; ^10^ Department of Molecular Genetics and Microbiology, Duke University, Durham, NC, United States; ^11^ Center for Virology and Vaccine Research, Beth Israel Deaconess Medical Center and Harvard Medical School, Boston, MA, United States

**Keywords:** NK cells, ADCC, Fc gamma receptor, antibody, FcR-mediated effector functions, rhesus macaques, principal component analysis

## Abstract

Rhesus macaques (RMs) are a common pre-clinical model used to test HIV vaccine efficacy and passive immunization strategies. Yet, it remains unclear to what extent the Fc-Fc receptor (FcR) interactions impacting antiviral activities of antibodies in RMs recapitulate those in humans. Here, we evaluated the FcR-related functionality of natural killer cells (NKs) from peripheral blood of uninfected humans and RMs to identify intra- and inter-species variation. NKs were screened for FcγRIIIa (human) and FcγRIII (RM) genotypes (FcγRIII(a)), receptor signaling, and antibody-dependent cellular cytotoxicity (ADCC), the latter mediated by a cocktail of monoclonal IgG1 antibodies with human or RM Fc. FcγRIII(a) genetic polymorphisms alone did not explain differences in NK effector functionality in either species cohort. Using the same parameters, hierarchical clustering separated each species into two clusters. Importantly, in principal components analyses, ADCC magnitude, NK contribution to ADCC, FcγRIII(a) cell-surface expression, and frequency of phosphorylated CD3ζ NK cells all contributed similarly to the first principal component within each species, demonstrating the importance of measuring multiple facets of NK cell function. Although ADCC potency was similar between species, we detected significant differences in frequencies of NK cells and pCD3ζ+ cells, level of cell-surface FcγRIII(a) expression, and NK-mediated ADCC (P<0.001), indicating that a combination of Fc-FcR parameters contribute to overall inter-species functional differences. These data strongly support the importance of multi-parameter analyses of Fc-FcR NK-mediated functions when evaluating efficacy of passive and active immunizations in pre- and clinical trials and identifying correlates of protection. The results also suggest that pre-screening animals for multiple FcR-mediated NK function would ensure even distribution of animals among treatment groups in future preclinical trials.

## 1 Introduction

Natural killer (NK) cells are critical for protection against, control, and clearance of human immunodeficiency virus type-1 (HIV-1) and simian immunodeficiency virus (SIV) infections (1–4). One of the important NK cell functions is antibody-mediated recognition and elimination of infected cells, known as antibody-dependent cellular cytotoxicity (ADCC). ADCC has been reported to play a role in lowering the risk of and protecting from HIV-1 infection in preclinical (5–7) and clinical studies (8–10) through recruitment of NK cells via the low affinity receptor that binds to the Fc portion of IgG, FcγRIIIa (CD16) or RM FcγRIII, the homologue for human FcγRIIIa. Throughout, we will use the nomenclature FcγRIII(a) when referring to both human FcγRIIIa and RM FcγRIII. The analysis of the FcγRIIIa allelic polymorphism in humans has revealed that a single nucleotide T to G substitution at position 559 in the second extracellular domain of FcγRIIIa results in an amino acid change from phenylalanine (F) at amino acid position 158 to valine (V) (11). The V/V, V/F, and F/F genotypes have been found to display different affinities for the IgG subclasses (12–15), with V/V resulting in the highest affinity for IgG1 and IgG3 (12). It has further been reported how these differences in FcγRIIIa polymorphisms influence predisposition to autoimmunity, impact cancer immunotherapy and infectious disease outcomes (13, 16–20). For instance, patients homozygous for the FcγRIIIa 158V respond better to treatment with low concentrations of anti-CD20 monoclonal Ab (mAb) rituximab in B cell malignancies (21), however, the difference in *in vitro* binding between different FcγRIIIa polymorphisms is lost with high concentrations of rituximab (22). Conversely, HIV-1-protein vaccinees homozygous for the FcγRIIIa V allele were more susceptible to HIV-1 infection than vaccine recipients with at least one F allele (23). It is important to note that FcγRIIIa genetic polymorphisms have not demonstrated reproducible association with HIV-1 acquisition, progression or vaccine efficacy, suggesting that further investigation of the impact of genetic variations on HIV-1 disease pathogenesis is needed (24).

NK cell functionality typically requires a net triggering of activating over inhibitory signals on the cell surface (25). Among the activation signals, triggering through FcγRIIIa can initiate the most robust signals for NK cell activation and even act as a mono-signal (26). NK cells do not, however, uniformly express FcγRIIIa and its various signaling adaptors. In direct relation to ADCC, previous research conducted by Shah et al. indicated that a subset of adaptive NK cells expresses FcγRIIIa but forgo the typical FcRγ/SYK pathway in favor of the CD3Ç heterodimer or homodimer and ZAP70, resulting in more potent antiviral function (27). Of note, this subset of NK cells, termed FcγRΔg NK cells, have been shown to exhibit enhanced ADCC against HIV/SIV, influenza, and HSV-1 antigens (27–30). Based on these data, we decided to focus our attention on the phosphorylation of CD3ζ as indicator of the activation pathways in NK cells.

Rhesus macaques (RMs) have been a fundamental animal model for the preclinical evaluation of HIV-1 candidate vaccines (31–34), but we are still missing data that can address the degree of similarities between humans and RMs related to NK cell-mediated immunity. Despite extensive testing of HIV-1 vaccine regimens in RMs, we have limited knowledge on the similarities that may exist in recapitulating human antibody Fc-FcR interactions in the RM model. Macaques express FcγRs that are significantly different in sequence, structure or expression profiles from humans (35–40). Although less investigated than human Fc receptors (FcRs), several studies have described genetic polymorphisms of rhesus macaque FcRs and have identified several variants not observed in humans (36, 41–43). However, the functional implications of this genetic variation remain incompletely defined. In fact, no evidence has been reported that genetic polymorphisms of RM FcγRIII may play a role in the protection from SIV/SHIV/HIV-1 infection observed in the pre-clinical studies conducted in RMs. According to our current knowledge, we hypothesize that these interspecies variations may result in substantial Fc-mediated functional differences and, therefore, need to be evaluated. Therefore, in this study we sought to evaluate the diversity of cellular biology factors that may predict Fc-FcR interactions and impact antibody dependent effector functions *in vivo.* We included FcγRIII(a) genotype as well as phenotypic and functional parameters of NK cell signaling significantly related to ADCC responses (27). We observed that the overall magnitude of ADCC had similar ranges in the two species. However, the frequencies of NK cells and phosphorylated CD3ζ (pCD3ζ+) cells, level of cell-surface FcγRIII(a) expression, and NK cell-mediated ADCC were significantly different between the species. In addition, total ADCC, pCD3ζ+ cells, level of cell-surface FcγRIII(a) expression, and NK cell-mediated ADCC contributed equally to intra-species variation in both humans and RMs. This study indicates that FcR-mediated NK cell functional outcomes are complex and should be analyzed in a multi-parametric approach. This approach will provide a better understanding of correlates of protection in clinical vaccine trials and allow for a proper randomization of animals in pre-clinical protection trials based on FcR-mediated NK functions.

## 2 Results

### 2.1 Comparison of FcR-related NK cell functional measurements between humans and rhesus macaques

**Figure 1:**
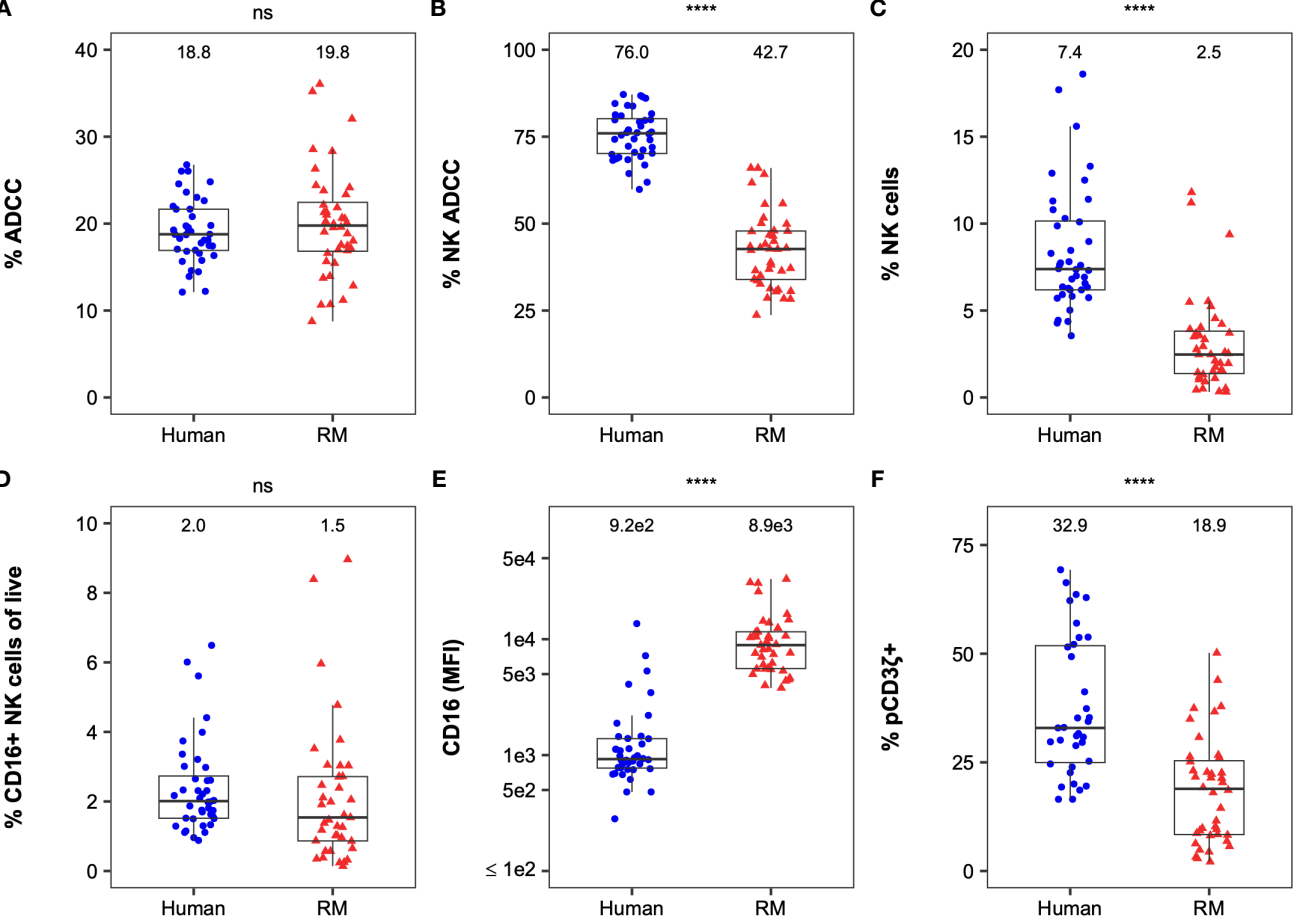
Comparison of FcR-related NK cell functional measurements between humans and RMs. Distribution of ADCC **(A)**, % NK-mediated ADCC **(B)**, % NK cells **(C)**, % CD16+ NK cellsof live cells **(D)**, CD16 expression, **(E)**, and %pCD3ζ+ **(F)** by species. Boxplots extend from 25^th^ percentile to 75^th^ percentile with a horizontal line at the median. Whiskers extend from the largest value within 25^th^ percentile + 1.5×IQR to the smallest value within 75^th^ percentile - 1.5×IQR, where the interquartile range (IQR) equals 75^th^ percentile - 25^th^ percentile. Medians are shown above the data. Each point represents an individual human or animal. N=40 humans (with the exception of %pCD3ζ+, for which N=35 humans) and N=40 RMs (with the exception of CD16, for which N=39 RMs). The statistical significance of species differences is reported as **** for P < 0.001, and “ns” for non-significant (P > 0.05) on the top of each panel and were calculated using two-sided Mann-Whitney *U* tests.

Peripheral blood mononuclear cells (PBMCs) from each human and rhesus macaque (RM) were analyzed for their effector functionality (**Figure 1**). A flow-based GranToxiLux (GTL) assay (44) was used to determine the ADCC magnitude of human or RM PBMCs. Target cells were coated with the recombinant SHIV1157 QNE Y173H gp120 protein for the GTL assay. To test ADCC potency, we used a combination of four HIV-specific human or RM IgG1 mAbs each at 1µg/ml (C1C2: JR4 (45, 46) and DH677.3 (45, 46); V1V2: DH827 (45, 47) and DH614.2 (45), which previously demonstrated binding to non-overlapping epitopes on gp120, and mediated ADCC against SHIV1157 QNE Y173H gp120-coated cells and SHIV1157 QNE Y173H-infected cells individually and in corresponding human and rhesus combinations (45, 48). We observed similar total ADCC activity across species (P > 0.05 by two-sided Mann-Whitney *U* test, **Figure 1A**). To assess the contribution of NK cells and monocytes to the total observed ADCC activity (% NK ADCC), we performed area scaling analysis (ASA, **Figure S1A**) (49). The contribution of NK cells to the total ADCC was significantly higher in humans compared to RMs (P < 0.001 by two-sided Mann-Whitney *U* test, **Figure 1B**) whereas NK cells contribution to the total ADCC was similar or lower than the monocyte contribution in RMs. The frequency of NK cells in PBMC samples was also significantly higher in humans compared to RMs (P < 0.001 by two-sided Mann-Whitney *U* test, **Figures 1C**, **S1B, C**). However, the frequency of CD16+ NK cells was similar across species (**Figure 1D**). We also analyzed the median fluorescent intensity (MFI) of cell-surface expressed FcγRIII(a) (CD16) within PBMCs, which is essential for ADCC by NK cells and monocytes (50, 51). Intriguingly, the level of cell-surface CD16 expression was significantly lower in humans (P < 0.001 by two-sided Mann-Whitney *U* test, **Figure 1E**). FcγRIII(a) expressed on NK cells can transduce signals through FcRγ and CD3ζ chains, where cells signaling through the alternative CD3ζ chain typically demonstrate enhanced ADCC (28, 52). Thus, we also measured the frequency of NK cells signaling through CD3ζ (phosphorylated CD3ζ; hereafter pCD3ζ) in PBMCs following FcγRIII(a) cross-linking (**Figure S1D**). The frequency of pCD3ζ+ NK cells was significantly higher in humans (P < 0.001 by two-sided Mann-Whitney *U* test, **Figure 1F**). Together, these data demonstrate variation in frequency and function of NK cells between the two species.

### 2.2 FcγRIII(a) genetic diversity as a predictor of FcR-related NK cell functions

Human PBMCs were genotyped for known polymorphisms of FcγRIIIa at amino acid position 158 (Phe or Val, F/V) in the membrane-proximal, IgG-binding domain, which demonstrated different affinity for antibody Fc with subsequent variation in ADCC potency (14). Among 40 human samples, we identified 17 samples with F/F genotype, 18 with F/V genotype and 5 with V/V genotype (**Table S1**). For RM cells, we were able to sequence *FCGR3* for 34 of the 40 animals (**Table S2**). Among those 34 RMs, 11 animals had no mutations relative to the reference sequence (Mnul_10, NCBI Genome ID: 215). One animal had only an I158V mutation, which is within the extracellular region of the receptor that forms the binding interface with IgG Fc (Tolbert et al., 2022, in press). 19 animals had both V211M and V215I mutations, which are located within the cytoplasmic tail. In addition, 3 animals had I158V, V211M, and V215I mutations (**Table S2**).

**Figure 2:**
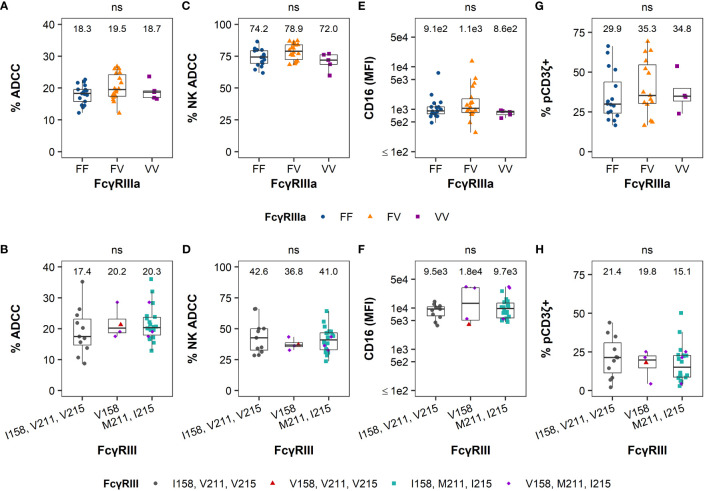
FcγRIII(a) genetic diversity is not a good predictor of FcR-related NK cell function. The distribution of ADCC potency (%ADCC), % NK-mediated ADCC, cell-surface FcγRIII(a) expression (CD16 MFI), and %pCD3ζ+ based on the human 158F/V polymorphism in humans are reported in the panels on the top row from the left to the right, respectively **(A, C, E, G)**. The distribution of the results for the same parameters based on the 158I/V, 211V/M, and 215V/I polymorphisms in RMs are reported in the panels on the bottom row in the same order **(B, D, F, H)**. The values for each parameter are reported on the y-axis and the polymorphisms on the x-axis. For simplicity and due to the low frequency of the I158V mutation in this cohort, all animals with the V158 allele are visualized together and all animals with M211 and I215 alleles are visualized together (with RMs with V158, M211, and I215 polymorphisms being represented twice in each of panels **(B, D, F, H)** rather than separating all 4 distinct haplotypes. Boxplots extend from 25^th^ percentile to 75^th^ percentile with a horizontal line at the median. Whiskers extend from the largest value within 25^th^ percentile + 1.5×IQR to the smallest value within 75^th^ percentile – 1.5×IQR, where the interquartile range (IQR) equals 75^th^ percentile – 25^th^ percentile. Medians are shown above each boxplot. Each point represents an individual human or animal; the color and shape represent the FcγRIIIa polymorphisms as indicated in the legend at the bottom of the panels). N=40 humans (with the exception of %pCD3ζ+, for which N=35 humans) and N=34 RMs. As indicated by “ns” at the top of each panel, no statistically significant differences in FcR-related NK functions were observed based on FcγRIII(a) polymorphisms among humans or RMs (all P > 0.05 using Kruskal-Wallis tests in **(A, C, E, G)** and Mann-Whitney *U* tests in **(B, D, F, H)**. In RM, comparisons of polymorphisms at each amino acid position were analyzed independently.

We then compared NK cell effector functions based on FcγRIII(a) polymorphisms to determine whether genetic diversity was predictive of functional diversity (**Figure 2**). The median ADCC magnitude was similar between genotypes in humans and RMs (**Figures 2A, B**). The contribution of NK cells and monocytes to the total observed ADCC activity was also similar among all genotypes in humans and RMs (**Figures 2C, D**). We observed similarity in cell-surface expression of FcγRIII(a) within PBMCs (**Figures 2E, F**) and the frequency of NK cells signaling through the CD3ζ chain (**Figures 2G, H**). Importantly, none of these measurements had a statistically significant association with FcγRIII(a) polymorphism(s) (all P > 0.05 by Kruskal-Wallis tests in humans and Mann-Whitney *U* tests in RMs, with comparisons of polymorphisms at each amino acid position being analyzed independently), suggesting that genetic diversity of FcγRIII(a) is insufficient to explain heterogeneity in NK cell function in either humans or macaques.

### 2.3 Functional data reveal clusters with distinct FcR-related NK functional profiles within each species

**Figure 3:**
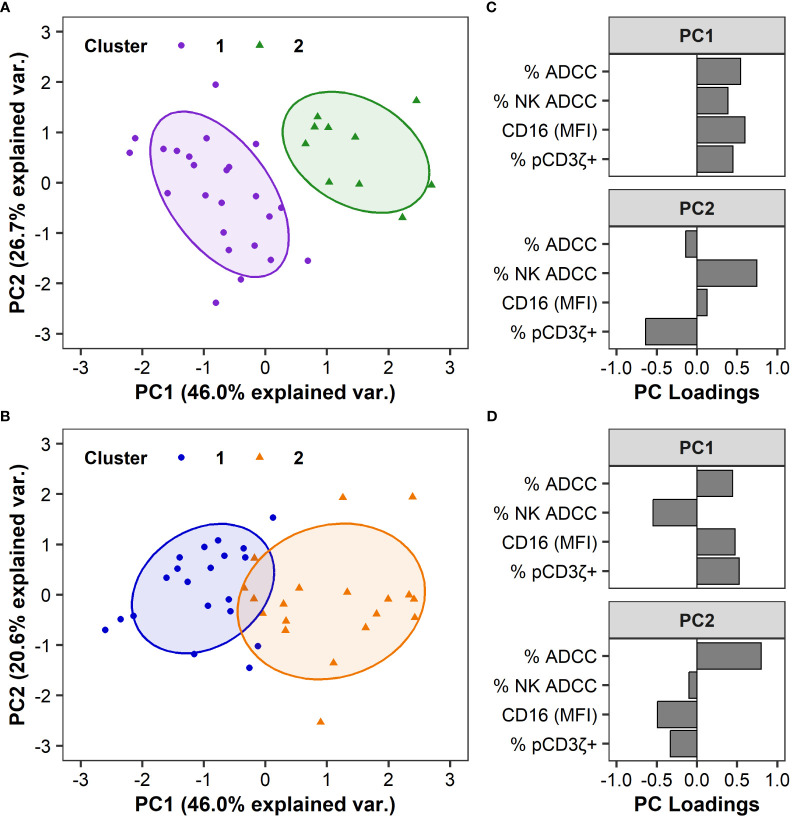
FcR-related NK cell functional measurements can separate humans and RMs into distinct clusters. Principal Component Analyses (PCA) are based on ADCC magnitude, % NK-mediated ADCC, CD16 MFI, or %pCD3ζ+ values. PCA biplot are shown by groups and identified using hierarchical clustering in humans **(A)** and RMs **(B)**, with ellipses showing 68% confidence area for each cluster. Each point represents an individual human or animal; clusters are color coded as indicated in the legend. Variable loadings for the first two principal components, PC1 and PC2, in humans **(C)** and RMs **(D)**. N=35 humans and N=39 RMs.

We observed heterogeneity among humans and RMs in each of the 4 functional measurements (**Figure 1**), yet this variability cannot be explained by FcγRIII(a) polymorphisms (**Figure 2**). Hierarchical clustering based on ADCC magnitude, % NK-mediated ADCC, cell-surface FcγRIII(a) expression (CD16 MFI), and % pCD3ζ+ identified 2 distinct groups within each species cohorts, which are separated on a principal component analysis (PCA) biplot for both humans (**Figure 3A**) and macaques (**Figure 3B**). In humans, F/F and V/V genotypes were overrepresented in cluster 1, while the F/V genotype was evenly balanced across clusters. However, the association between polymorphism and cluster was not statistically significant (P = 0.051 by Fisher’s Exact test, **Table 1**). In RMs, all polymorphisms were represented in each cluster and there were no significant associations between polymorphisms and clusters (P > 0.05 by Fisher’s Exact test, **Table 1**). In humans, the clusters separated well by principal component 1 (PC1), which explained 46.0% of the total variation within the cohort. All four parameters contributed similarly to PC1 (**Figure 3C**). The largest contributors to PC2, which explained an additional 26.7% of the total variation, were % NK-mediated ADCC and % pCD3ζ+ NK cells. In RMs, PC1 explained 46.0% of the total variation within the cohort and the magnitude of contributions to PC1 were similar for all four parameters, consistent with results in the human cohort, but the loading value for % NK ADCC was negative while loadings for the other 3 parameters were positive (**Figure 3D**). The largest contributor to PC2, which explained an additional 20.6% of the total variation, was the total ADCC potency.

**Table 1:** FcγRIII(a) polymorphism(s) by cluster in humans and rhesus macaques.

Species	FcγRIII(a) polymorphism	Alleles	Cluster 1	Cluster 2	P-value
**Human**	F158V	F/F	13	3	0.051
		F/V	7	8	
		V/V	4	0	
**RM**	I158V	I/I	15	15	1.000
		I/V	2	2	
	V211M	V/V	7	5	0.721
		V/M or M/M	10	12	
	V215I	V/V	7	5	0.721
		V/I or I/I	10	12	

Associations between FcγRIII(a) polymorphisms and cluster were tested using Fisher’s Exact test.

**Figure 4:**
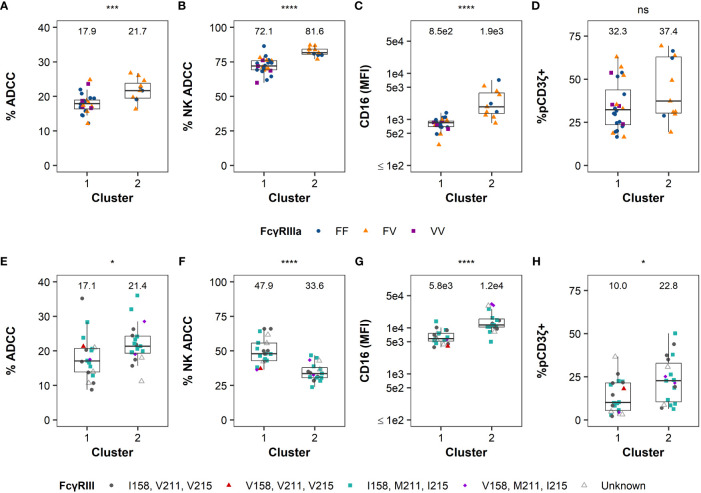
FcR-related NK cell functional measurements by cluster. Distribution of ADCC magnitude, % NK-mediated ADCC, CD16 expression, and %pCD3ζ+ by groups identified using hierarchical clustering are reported in the panels left to right, respectively, in humans **(A–D)** or RMs **(E–H)**. Boxplots extend from 25^th^ percentile to 75^th^ percentile with a horizontal line at the median. Whiskers extend from the largest value within 25^th^ percentile + 1.5×IQR to the smallest value within 75^th^ percentile - 1.5×IQR, where the interquartile range (IQR) equals 75^th^ percentile - 25^th^ percentile. Data from each individual human and animal shown as a point, with color and shape by FcγRIII(a) polymorphism(s). RMs labeled as unknown if haplotype was not determined. Medians are shown above the data. N=35 humans and N=39 RMs. The statistical significance of the differences is indicated on the top of each panel as * for P < 0.05, ** for P < 0.01, *** for P < 0.005, **** for P < 0.001, and “ns” for non-significant (P > 0.05), and calculated by two-sided Mann-Whitney *U* tests.

The functional profiles of each cluster were further characterized by examining the distribution of the variables by cluster (**Figure 4**). For human samples, we observed significant differences in ADCC potency (P = 0.002, **Figure 4A**), % NK ADCC (P < 0.001, **Figure 4B**), and cell-surface FcγRIIIa expression CD16 (MFI; P < 0.001, **Figure 3C**) between clusters by Mann-Whitney *U* tests, with medians for all parameters being higher in cluster 2. Median % pCD3ζ+ was also higher in cluster 2, however there was no statistically significant difference between clusters (P > 0.05 by Mann-Whitney *U* test, **Figure 4D**).

For RM samples, there were significant differences between clusters in all four parameters (by Mann-Whitney *U* tests) where ADCC potency (P = 0.016, **Figure 4E**), CD16 MFI (P < 0.001, **Figure 4G**), and % pCD3ζ (P = 0.013, **Figure 4H**) were higher in cluster 2, while median % NK ADCC was higher in cluster 1. Altogether, these data indicate that human subjects and RMs can be separated into clusters with distinct functional profiles. Clustering of RMs is independent of FcγRIII genetic diversity, and while there is some evidence that clustering of humans could be influenced by FcγRIIIa polymorphisms, the association was not statistically significant.

Spearman’s rank correlation was used to examine relationships among these functional measurements within humans (**Figure S2**) and RMs (**Figure S3**). In humans, no significant correlations were observed to frequency of NK cells (P > 0.05, **Figures S2A–D**), but positive correlations were observed between total ADCC and CD16 expression (r = 0.465, P = 0.002, **Figure S2F**), ADCC and the frequency of pCD3ζ+ NK cells (r = 0.425, P = 0.011, **Figure S2G**), and frequency of NK-cell mediated ADCC and CD16 expression (r = 0.414, P = 0.008, **Figure S2H**). In RMs, the frequency of NK cells had a negative correlation to total ADCC potency (r = -0.344, P = 0.032, **Figure S2A**) and a positive correlation to NK-cell mediated ADCC (r = 0.538, P < 0.001, **Figure S3B**). NK-cell mediated ADCC had negative correlation to the total ADCC potency (r = -0.435, P = 0.005, **Figure S3E**), CD16 expression (r = -0.383, P = 0.016, **Figure S3H**), and the frequency of pCD3ζ+ NK cells (r = -0.411, P = 0.009, **Figure S3I**). CD16 expression had positive correlation to the frequency of pCD3ζ+ NK cells (r = 0.387, P = 0.015, **Figure S3J**). These weak to moderate correlations, although they reached statistical significance, highlight that no single parameter alone is sufficient to characterize these different aspects of NK cell function.

### 2.4 Cohort demographics

The human cohort presented here consisted of 24 males and 16 females with CMV status available for 38 individuals (**Table S1**), while all 40 RM were CMV+ males (**Table S2**). Based on descriptive statistics we observed little evidence of any NK cell functional differences based on gender or CMV status in this human cohort (**Figure S4**).

### 2.5 Randomizing animals for groups with balanced NK cell functions

**Figure 5:**
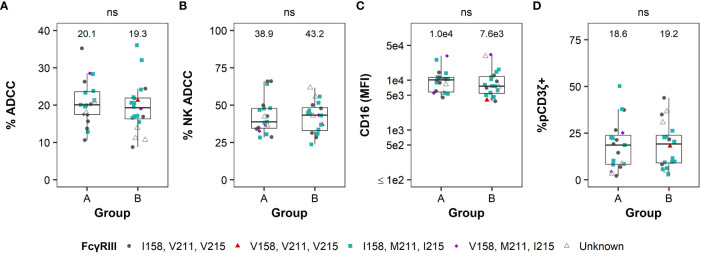
Covariate constrained randomization of RMs produced balanced groups for FcR-related NK cell functional measurements. Distribution of ADCC magnitude **(A)**, % NK-mediated ADCC **(B)**, CD16 MFI **(C)**, and %pCD3ζ+ **(D)** according to group assignments based on covariate constrained randomization (see Methods for details). Boxplots extend from 25^th^ percentile to 75^th^ percentile with a horizontal line at the median. Whiskers extend from the largest value within 25^th^ percentile + 1.5×IQR to the smallest value within 75^th^ percentile - 1.5×IQR, where the interquartile range (IQR) equals 75^th^ percentile - 25^th^ percentile. Data from each individual animal shown as a point, with color and shape by FcγRIII polymorphisms. RMs labeled as unknown if haplotype was not determined. Medians are shown above the data. N=39. No statistically significant differences in FcR-related NK functions were observed between groups A and B (all P > 0.05 by two-sided Mann-Whitney *U* tests).

Based on the heterogeneity observed in FcR-related NK cell functional measurements within these cohorts, we sought to investigate whether we could use screening data to assign treatment groups of RMs in pre-clinical trials to ensure balanced groups. We used covariate constrained randomization to assign animals into the potential treatment groups based on the four functional parameters (**Figure 5**). We observed that potential treatment groups A and B showed similar % total ADCC (**Figure 5A**), contribution of NK to the total ADCC (**Figure 5B**), CD16 expression (**Figure 5C**), and frequency of pCD3ζ+ NK cells (**Figure 5D**) with no significant differences (P > 0.05 by Mann-Whitney *U* test). Together, these data demonstrate that assigning RM to treatment groups using covariate constrained randomization produced balanced groups with similar FcR-related NK cell functional profiles.

We further sought to investigate whether covariate constrained randomization of animals will introduce bias on non-NK functional parameters, such as antibody-dependent cellular phagocytosis (ADCP). ADCP has been demonstrated to contribute to protection against infection in preclinical (7, 53, 54) and clinical studies (55). We compared phagocytic activity of monocytes, isolated from RM PBMCs, as defined by a phagocytosis score–the mathematical product of the percentage of phagocytic-active cells and the number of phagocytosed particles per cell (56). Although phagocytosis occurs independently of FcγRIII, we analyzed whether FcγRIII polymorphisms or the NK functional clusters identified in **Figure 2** were predictive of *in vitro* phagocytosis. There were no significant differences between FcγRIII polymorphism groups or NK functional clusters for RMs (P > 0.05 by Kruskal-Wallis test, **Figures S5A, B**). Importantly, covariate constrained randomization of RMs showed similar ADCP score in groups A and B with similar ranges and no significant difference (P > 0.05 by Mann-Whitney *U* test, **Figure S5C**). These data demonstrate that assigning RMs into treatment groups using covariate constrained randomization produced groups with similar NK cell functional profiles without introducing bias in non-NK functions, such as ADCP.

## 3 Discussion

The first clinical trial with administration of monoclonal Ab to treat HIV-1 was introduced in 1994 (57). Today, 36 clinical trials with administration of neutralizing Abs for prevention and treatment of HIV-1 have been launched (58, 59). Both pre-clinical studies (7, 54, 60–65) and clinical trials (55, 66) have demonstrated that Fc-mediated Ab functions contribute to the protection against infection and control of disease progression provided by neutralizing Abs. In addition, the only clinical trial that has shown limited efficacy (RV144) indicated that most of the anti-HIV antibody responses generated by infected individuals did not display neutralizing activities but contributed to protection by mediating ADCC against HIV-infected cells in vaccine recipients with low anti-HIV-1 IgA responses (8, 10). Therefore, these studies suggest the crucial relevance of evaluating Fc-FcR mediated functions as a correlate of risk and/or protection in pre- and clinical trials.

Our study represents the first attempt to address functional, phenotypic, and genetic diversity of effector NK cells among human and RM samples and compare diversity between the two species. Specifically, we analyzed NK cell phenotypic and the extent to which FcγRIII(a) polymorphisms influence the heterogeneity of NK-dependent functional responses. Multivariate analysis results indicated that *FCGR3* genotype alone is not sufficient to explain this heterogeneity in human or RM samples. Animals and humans can be clustered into groups with distinct functional profiles; however, clusters were not fully explained by FcγRIII(a) polymorphisms alone. Instead, a combination of four FcR-related NK-cell parameters tested here contributed to the functional differences observed in both human and RM cohorts.

While FcγRIIIa polymorphisms in human NK cells have been linked to treatment efficacies and disease outcomes in many settings (16–20), the effect of rhesus macaque FcγRIII polymorphisms on study outcomes has largely been understudied. Here, we investigated whether genetic polymorphism in FcγRIII had an impact on rhesus macaque FcR-related NK cell functions *in vitro*. We identified polymorphisms at three sites (I158V, V211M, and V215I) and found that none of these polymorphisms were associated with the identified NK cell functional clusters, nor with any of the individual NK cell functions tested in our study. Previous work demonstrated that rhesus FcγRIII I158 has slightly higher binding affinity for rhesus macaque IgG1 compared to V158 variant (43, 67), but that these differences can vary based on the glycan composition of the FcR and Fc of Ab. Our data is consistent with these results demonstrating that FcγRIII(a) genotype alone cannot predict NK functional differences in either of the species. Our findings are also consistent with recent work demonstrating that polymorphisms at position 158, 211, and 215 of rhesus FcγRIII do not significantly impact ADCC mediated by rhesus IgG1 antibodies (42). However, the abundance of cell-surface FcγRIII can increase ADCC potency of RM NK cells even if NK cells express lower affinity FcγRIII polymorphism (67). Collectively, these observations indicate that rhesus NK cells expressing common allotypes of FcγRIII have similar functionality *in vitro* and the combination of FcR-related parameters influence functional differences. It is important to note however, that in our study, functional clusters of human NK cells were also not associated with FcγRIIIa SNPs. This *in vitro* finding is in stark contrast to the numerous *in vivo* associations linking SNPs and position 158 with human clinical outcomes (16–20). Therefore, although we observed no statistical differences in function and phenotype of rhesus macaque NK cells based on FcγRIII genetic diversity *in vitro*, it remains possible that these polymorphisms may have *in vivo* effects that are not yet known.

We observed that on average human PBMCs have higher frequencies of NK cells compared to RM PBMCs, whereas cell-surface FcγRIIIa expression (CD16) on human NK cells was significantly lower than CD16 on RM NK cells. Despite the differences in CD16 MFI between humans and RMs, CD16 expression was neither significantly different among SNPs in humans or RMs, nor did it contribute to functional clustering in humans or RMs. Interestingly, the contribution of NK to ADCC was higher in human samples compared to RMs. These dissimilarities are related not only to the cell-surface expression of FcγRIII(a) but also to the difference in binding affinity of human Ab Fc to FcγRIIIa with different polymorphisms vs RM counterparts (43, 67, 68). Here, for simplicity, we generated all mAbs with either human IgG1 or RM IgG1 Fc. Human FcγRIIIa has the highest affinity to IgG1 compared to other isotypes. Human IgG2 binds weakly to FcγRIIIa (15) and is not effective at recruiting NK cells for ADCC (69). Human IgG3 isotype Abs have longer hinge region, better flexibility, and mostly contribute to neutralization and ADCP responses (70). NHP IgG3 Abs have shorter hinge region and have similar binding affinity to FcγRIII and comparable functionality to human IgG1, NHP IgG1 and IgG2 (40, 71). Since the focus of this study is the ADCC-mediated activity of NK cells through Ab Fc-NK FcγRIIIa interaction and did not include evaluation of binding affinities between Fcs and FcRs with different polymorphisms, we chose to produce mAbs with IgG1 Fc for the most optimal ADCC activity.

CD16 does still require physically associated transmembrane adaptors for subsequent signal transduction and typically associates with FcγR and its adaptor, SYK (72). Interestingly, in primates (including humans), CD16 can also utilize CD3Ç in lieu of FcRγ chain, which presents as a disulfide-linked homodimer or as a heterodimer with FcRγ (72). Upon experimental cross-linking of CD16 or antibody ligation, immunoreceptor tyrosine-based activation motifs (ITAMs) on these adaptors are phosphorylated and act as docking sites for the cytoplasmic kinases SYK and ZAP70, which in turn activate downstream signaling pathways inducing ADCC (72). Importantly, NK cell signaling through CD3Ç results in higher ADCC functionality compared to canonical γ-chain (27, 73), these observations drove our decision to focus on this signaling pathway to understand the diversity of Fc-FcR interaction in human and RMs. In our study, we observed that subsets of both human and RM NK cells use CD3ζ/Zap70 signaling, but a higher frequency of human NK cells signal through CD3ζ compared to RMs; however, in both species the level of signaling neither correlates nor cluster with the FcγRIII(a) polymorphisms.

We decided to use covariate constrained randomization to separate animals into two groups equally balanced for Fc-mediated NK functions by using the combination of four FcγRIII-related phenotypic and functional parameters, such as total ADCC, CD16 MFI, %pCD3ζ and %NK ADCC. It should be noted, randomization of animals into two groups based on Fc-FcR related parameters did not introduce bias in non-NK cell functions. While animals with protective alleles (Mamu-B*17+ and B*08+) are excluded from pre-clinical trials (74), we do not suggest the exclusion of animals based on polymorphisms, but propose that screening animals for FcR-mediated NK cell functions will be useful prior to initiating pre-clinical studies, which often have small sample sizes, to ensure even balance in these measures among study arms. Here we used only males rhesus macaques, however, both sexes should be considered to ensure there are no additional gender-related variability that may contribute to NK functionality. Prospective interrogation of RM FcR-related functional parameters will ensure correct randomization of Rhesus macaques in pre-clinical studies intended to evaluate correlates of risk and/or protection, while retrospective multivariate analysis of human Fc-mediated NK functions in autologous systems utilizing Abs and NK cells from the same participants may lead to a better understanding of correlates of risk and/or protection in vaccine and passive infusion efficacy clinical trial.

We have not yet been able to analyze samples collected from humans and RMs who were vaccinated or used in pre-clinical efficacy studies, respectively, but this study indicates that FcR-mediated NK cell functional outcomes are complex and should be analyzed in a multi-parametric approach. In this study we focused primarily on F158V polymorphism of human NK cells which is known to affect affinity for the IgG subclasses and directly impact NK-mediated ADCC. Additional genetic variations should be explored as potential contributors to NK functionality. Additionally, we did not evaluate copy number of FCGR3A alleles that also were reported to affiliate with FcγRIIIa expression on NK cells (75). Future studies including NK functionality should consider including these parameters.

In conclusion, our studies strongly demonstrated that Fc-FcR interaction in pre- and clinical studies cannot be evaluated by simple analysis of the genetic polymorphisms but need multiparameter in-depth functional analyses of FcR-related NK functions in order to link efficacies of passive and active immunization, correlates of protection in pre- and clinical trials, and to ensure even distribution of treatment groups.

## 4 Materials and methods

### 4.1 Ethics statement

The studies were reviewed and approved by the Duke University Medical Center Institutional Review Board, and all participants provided written informed consent. All research was performed in accordance with the Duke University School of Medicine guidelines and regulations. Indian origin rhesus macaques (*Macaca mulatta*) were used in this study. The animals were housed at the New Iberia Research Center, animal experiments were approved by the New Iberia Primate Research Center IACUC (Protocol 2021-010-8823) and conducted in compliance with the principles described in the *Guide for the Care and Use of Laboratory Animals* (76). Animals with B*17+ and B*08+ alleles were excluded from this cohort due to the protective effect of these alleles (74). Human samples were collected at and experiments with human samples were approved by the Duke IRB (Pro00000873).

### 4.2 Human Fc Receptor sequence analysis

FcγRIIIa is encoded by the gene *FCGR3A.* Genotyping of the donors for SNP rs396991 was determined by TaqMan SNP Genotyping Pre-Validated Assay (Applied Biosystems, Foster City, CA) and an ABI7900 Sequence Detection System (ABI). The genotyping assay was performed according to manufacturer’s guidelines using 10 ng of DNA. Standard TaqMan thermocycling conditions were 10 min at 95°C, then 40 cycles of 15 s at 92°C and 1 min at 60°C. The endpoint fluorescence was read on an ABI 7900 Sequence Analyzer. The allele call was made by using the ABI proprietary software SDS version 2.4.

### 4.3 Rhesus macaque Fc receptor sequence analysis

*FCGR3* sequencing was performed using long-read RNA sequencing as previously described (PMID: 34093580). Briefly, RNA was isolated from RM PBMC samples using the AllPrep DNA/RNA isolation kit (Qiagen, Germantown, MD), reverse transcribed using the Qiagen QuantiTect Reverse Transcription Kit (Qiagen), and Fc receptor cDNA was amplified using gene-specific primers designed with PacBio barcodes (Pacific Biosciences, Menlo Park, CA). PCR products were purified and PacBio SMRTbell library preparation was performed in accordance with manufacturer’s recommendations (Pacific Biosciences). Sequencing was performed on a PacBio Sequel II instrument using 2.1 or 3.0 chemistry (Pacific Biosciences). Datasets were loaded into PacBio SMRT Link 7.0.1 software package for demultiplexing of subreads and generating circular consensus sequences (CCS) and data analysis was conducted using a pipeline similar to that previously described (77). *FCGR3* sequences were aligned to Mmul_10 reference sequence (NCBI Genome ID: 215) using a long-read sequence alignment tool (78) and variants were identified using the GATK Haplotype Caller (79), and annotated with ANNOVAR (80).

### 4.4 Monoclonal antibody production

Human IgG1 and RM IgG1 mAbs were produced by transient transfection of heavy and light chain plasmids into Expi293-F cells with Expifectamine (Thermo Fisher Scientific, Waltham, MA) and purified from cell culture supernatants by protein A resin columns as previously described (81–83).

### 4.5 HIV-1 Env gp120-specific ADCC

The ADCC-GranToxiLux assay was used to measure the ability of immune effector cells present in rhesus macaque (RM) PBMC to mediate Env gp120-specific ADCC when directed by HIV-specific RM IgG1 monoclonal antibodies. The assay was performed as previously described (44, 49). Target cells were a clonal isolate of the CEM.NKR_CCR5_ CD4^+^ T cell line (NIH AIDS Reagent Program, Division of AIDS, NIAID, NIH: from Dr. Alexandra Trkola (84)) coated with SHIV1157 QNE Y173H gp120 protein (7). Human or RM PBMC effector cells were used at an effector cell to target cell ratio of 30:1 or 60:1, respectively. The antibodies used were a combination of the C1C2 cluster A-region specific antibodies JR4 (85) and DH677.3 (46), and V1V2-specific antibodies DH614.2 (86) and DH827 (47). JR4 and DH614.2 were originally isolated from RM after SHIV-infection or HIV-vaccination, respectively. DH677.3 and DH827 were originally isolated from human volunteers in the RV306 clinical trial. All antibodies were recombinantly produced as human and rhesus IgG1 (82). The combination of four human or four rhesus mAb were tested at a concentration of 1 μg/ml each. The influenza specific human or rhesusized CH65-IgG1 (anti-HA) antibodies (87) were used at the equivalent concentration of 4 μg/mL as negative controls. Data were reported as the maximum proportion of cells positive for proteolytically active granzyme B (GzB) out of the total viable target cell population (maximum %GzB activity) after subtracting the background activity observed in wells containing effector and target cells in the absence of antibodies. Area scaling analysis (ASA) of the GzB^+^ cells was used to evaluate antibody-dependent recruitment of NK cells (% NK ADCC) and monocytes as previously described (49).

### 4.6 NK cell frequency and expression of cell-surface CD16

Flow cytometry was used to assess the frequency of NK cells in human and RM PBMC, and to measure the levels of cell surface CD16. Cryopreserved PBMC were thawed and incubated overnight (18 hours) in RPMI1640 medium supplemented with 10% FBS at 37°C, 5% CO_2_. The cells were then washed with PBS and stained with a viability marker (Fixable Aqua Dead Cell Stain Kit, Thermo Fisher Scientific, San Diego, CA) prior to surface staining with fluorescently conjugated monoclonal antibodies using standard techniques. The panels of fluorescently conjugated antibodies for NK cell phenotyping were modified from what previously published (88, 89). Rhesus PBMCs were phenotyped with: CD3 (Pacific Blue, clone SP34.2, BD Biosciences), CD20 (Alexa Fluor 700, clone 2H7, BD Biosciences), CD8 (APC-H7, clone SK1, BD Biosciences), NKG2A/C (CD159a/c; PE, clone Z199, Beckman-Coulter), CD14 (BV650, clone M5E2, BD Biosciences), CD16 (PE-CF594, clone 3G8, BD Biosciences). Human PBMCs were phenotyped with: CD3 (PE-CF594, clone S4.1/7D6, ThermoFisher), CD4 (PerCPCy5.5, clone OKT4, eBioscience), CD8 (BV650, clone RPA-T8, Biolegend), NKG2A/C (CD159a/c; PE, clone Z199, Beckman-Coulter), CD14 (PE-Cy5, clone Tuk4, Life Technologies), CD19 (PE-Cy5, clone SJ25-C, Invitrogen), CD16 (APC-Cy7, clone 3G8, Beckman Coulter), CD56 (PE-Cy7, clone NCAM16.2, BD Biosciences). 

Data analyses were performed using FlowJo software (v10.5.3). RM NK cells were defined as live CD3^-^ CD14^-^ CD20^-^ CD8^+^ NKG2A/C^+^, as previously described (90). CD16 expression for human and RM cells is represented by APC-Cy7 or PE-CF594, respectively, median fluorescence intensity (MFI) of the CD16^+^ NK cell population. All samples were analyzed on a BD LSRFortessa flow cytometer (BD Biosciences) that has been optimized and rigorously maintained under quality control procedures described by Perfetto and colleagues (91).

### 4.7 Phospho-flow

Phosphorylation of CD3ζ chain (pCD3ζ) was determined as previously described (27). BD Perm buffer III (BD Biosciences, La Jolla, CA) was used for phospho-flow analyses according to the manufacturers’ protocols. Briefly, cells were opsonized with anti-human CD16 (BD Biosciences, clone 3G8, La Jolla, CA) followed by cross-linking with secondary goat-anti-mouse F(ab)’2, (Jackson ImmunoResearch Laboratories, West Grove, PA). Reactions were immediately stopped after 2.5 minutes (experimentally determined, data not shown) with equal volumes of Fix Buffer I (BD Biosciences, La Jolla, CA). After washing, unused/unoccupied sites of the secondary antibody were blocked with normal mouse serum (ThermoFisher) for 10 minutes at room temperature. Fixation was followed by surface stain, permeabilization with Perm Buffer III for 30 minutes on ice, followed by intracellular staining at room temperature according to the manufacturer’s protocol.

### 4.8 Antibody dependent cellular phagocytosis

ADCP was performed as previously described (56, 92). Briefly, quantification of ADCP was performed by covalently binding SHIV1157 QNE Y173H gp120 protein (7) to fluorescent beads and forming immune complexes by incubating in the presence of combination of four rhesus mAbs each at 25 µg/mL. Immune complexes were then incubated in the presence of rhesus monocytes isolated (Miltenyi Biotec catalog #130-091-097) from cryo-preserved rhesus PBMCs rested overnight (18 hours) in RPMI1640 medium supplemented with 10% FBS at 37°C, 5% CO_2_ and then washed with PBS. Fluorescence of the cells was detected using flow cytometry (BD Fortessa cytometer). The magnitude of the ADCP immune response was calculated as an ADCP score by multiplying the mean fluorescence intensity (MFI) and frequency of phagocytosis-positive cells and dividing by the MFI and frequency of the bead-positive cells in an antibody-negative (PBS) control well. The influenza specific rhesusized CH65-IgG1 (anti-HA) antibody (87) at the equivalent concentration of 100 μg/mL was used as a negative control. Each sample was tested once, and ADCP score is the mean score from two biological replicates.

### 4.9 Statistical analysis

Statistical analyses were performed using R statistical software (93). Clusters were identified using Ward’s hierarchical clustering based on Euclidian distance. Clustering was performed using ADCC, % NK ADCC, % pCD3ζ+, and CD16 expression data with and without the available demographic variables gender, age, race, weight and CMV status. However, these demographic variables did not impact the clustering results in humans or RMs. Principal components analysis was performed using the same 4 functional measurements that went into the cluster analysis and visualized using the ‘factoextra’ package (94). Five humans were missing % pCD3ζ+ values and one RM was missing a CD16 expression value; these subjects were excluded from clustering and PCA. Prior to clustering and PCA, CD16 expression values were log-transformed, and then ADCC, % NK ADCC, % pCD3ζ+, and CD16 were each scaled to have mean 0 and standard deviation 1 within each species cohort. To obtain the groups shown in **Figure 5**, we used covariate constrained randomization to assign the potential treatment arms based on the four variables discussed. The randomization was done using the R package cvcrand (95), examining 50,000 potential randomization schemes with the potential assignment sampled from the lowest 10% of the resulting inverse variance weighted balance scores (96).

Kruskal-Wallis tests were used when there were more than 2 groups for comparison (**Figure 4**). Two-sided Mann-Whitney *U* tests were used for pairwise comparisons (**Figures 1**, **4**, **5**). Two-sided Fisher’s exact tests were used to test for association of categorical variables (**Table 1**). All analyses were performed separately by species, except for the direct comparison of humans and macaques shown in **Figure 1**. All comparisons were considered significant at the 0.05 level. Due to the exploratory nature of these analyses, no corrections were made for multiple testing.

## Data availability statement

The RM sequencing data presented in the study are deposited in The National Center for Biotechnology Information (NCBI) repository, under the umbrella BioProject PRJNA1030012. RM demographic and functional data are available for download at doi.org/10.5281/zenodo.8436837. Human data is available upon request.

## Ethics statement

The studies involving humans were approved by Duke University Medical Center Institutional Review Board. The studies were conducted in accordance with the local legislation and institutional requirements. The participants provided their written informed consent to participate in this study. The animal study was approved by Duke University Medical Center Institutional Review Board. The study was conducted in accordance with the local legislation and institutional requirements.

## Author contributions

MT: Conceptualization, Methodology, Writing – original draft, Writing – review & editing. RS: Conceptualization, Formal Analysis, Writing – original draft, Writing – review & editing. BH: Methodology, Writing – review & editing. JN: Methodology, Writing – review & editing. DG: Methodology, Writing – review & editing. CC: Formal Analysis, Supervision, Writing – review & editing. RB: Formal Analysis, Methodology, Writing – review & editing. WB: Methodology, Writing – review & editing. SJ: Formal Analysis, Writing – review & editing. SA: Methodology, Writing – review & editing. KW: Methodology, Writing – review & editing. MH: Methodology, Writing – review & editing. DE: Methodology, Writing – review & editing. HC: Methodology, Writing – review & editing. TH: Methodology, Writing – review & editing. TG: Methodology, Writing – review & editing. CJ: Methodology, Writing – review & editing. NA: Methodology. FV: Resources, Writing – review & editing. RT: Methodology, Writing – review & editing. TD: Writing – review & editing, Resources. MM: Methodology, Supervision, Writing – review & editing. GT: Funding acquisition, Resources, Supervision, Writing – review & editing. RR: Conceptualization, Funding acquisition, Resources, Supervision, Writing – original draft. JP: Conceptualization, Funding acquisition, Resources, Writing – original draft. GF: Conceptualization, Funding acquisition, Resources, Supervision, Writing – original draft.
