# Supplementary material for: Multivariate analysis of FcR-mediated NK cell functions identifies unique clustering among humans and rhesus macaques
Source: Front Immunol. 2023 Dec 6;14:1260377. doi: 10.3389/fimmu.2023.1260377 (PMC10732150; doi:10.3389/fimmu.2023.1260377)
Supplement: Supplementary file 1 [file DataSheet_1.pdf]

Supplementary Material

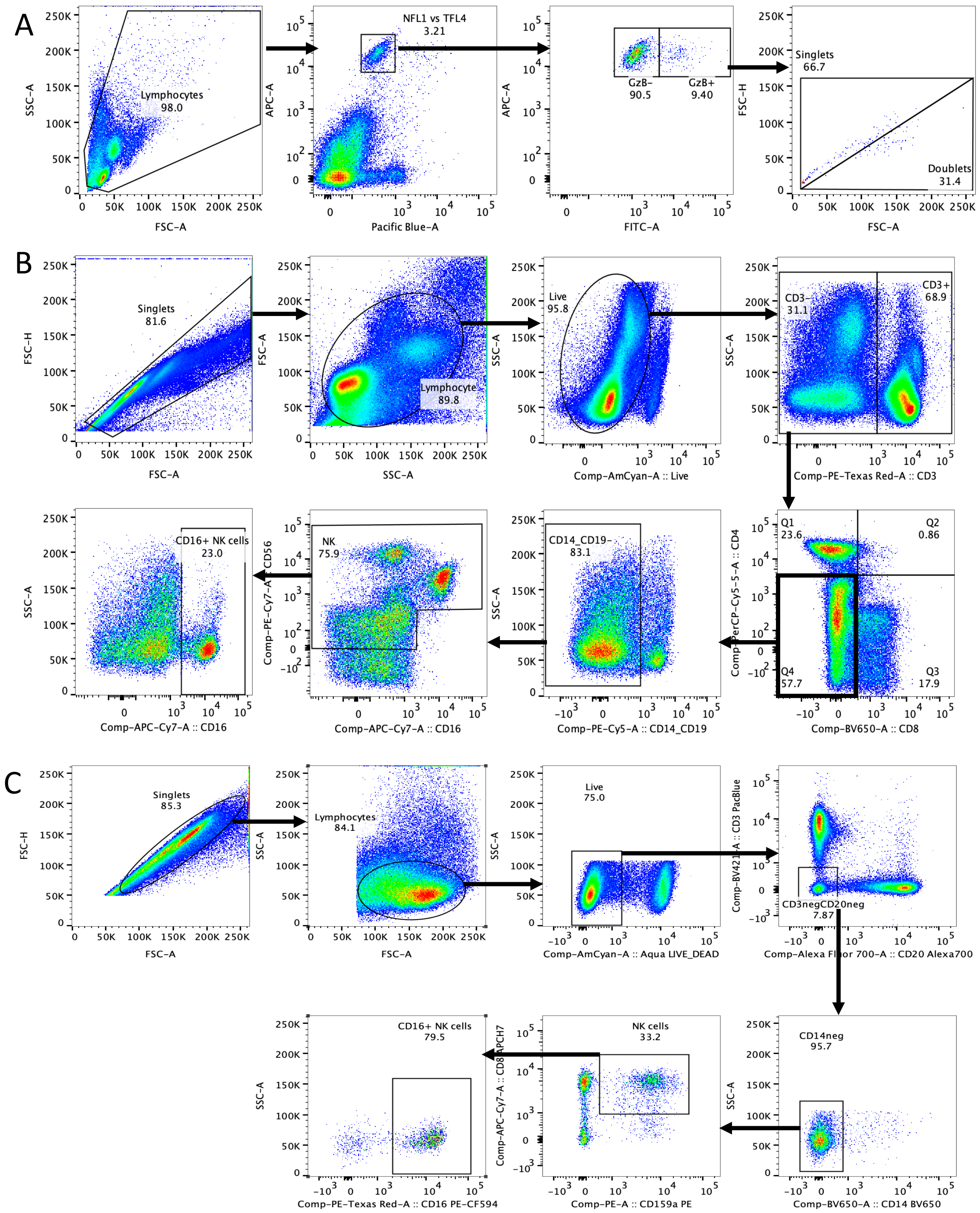

Figure S1. Gating strategy. (A) GranToxiLux (GTL) assay. Using gp120-coated target cells alone, the first gate was set to include total target cells. The identification of the viable target cells is performed by combining the analysis of the signal emitted by the TFL4 (separation of target from effector cells) and NFL1 (separation of viable NFL1 negative from dead NFL1 positive cells) as reported in the next dot plots. The GzB positive cells are identified as those that are outside the GzB negative gate set using the gate from the target in absence of the effectors. The frequency of the GzB positive population in the condition where target and effector cells were incubated without antibodies was considered as our background activity. Application of Area Scaling Analysis (ASA) was used to identify singlet (and non-singlet (doublets) GzB+ target cell events. (**B**) Gating strategy for human CD3-CD4-CD8-CD14-CD19-CD56+CD16+ NK cells (**C**) Gating strategy for rhesus CD3-CD20-CD14-CD8+CD159a+CD16+ NK cells

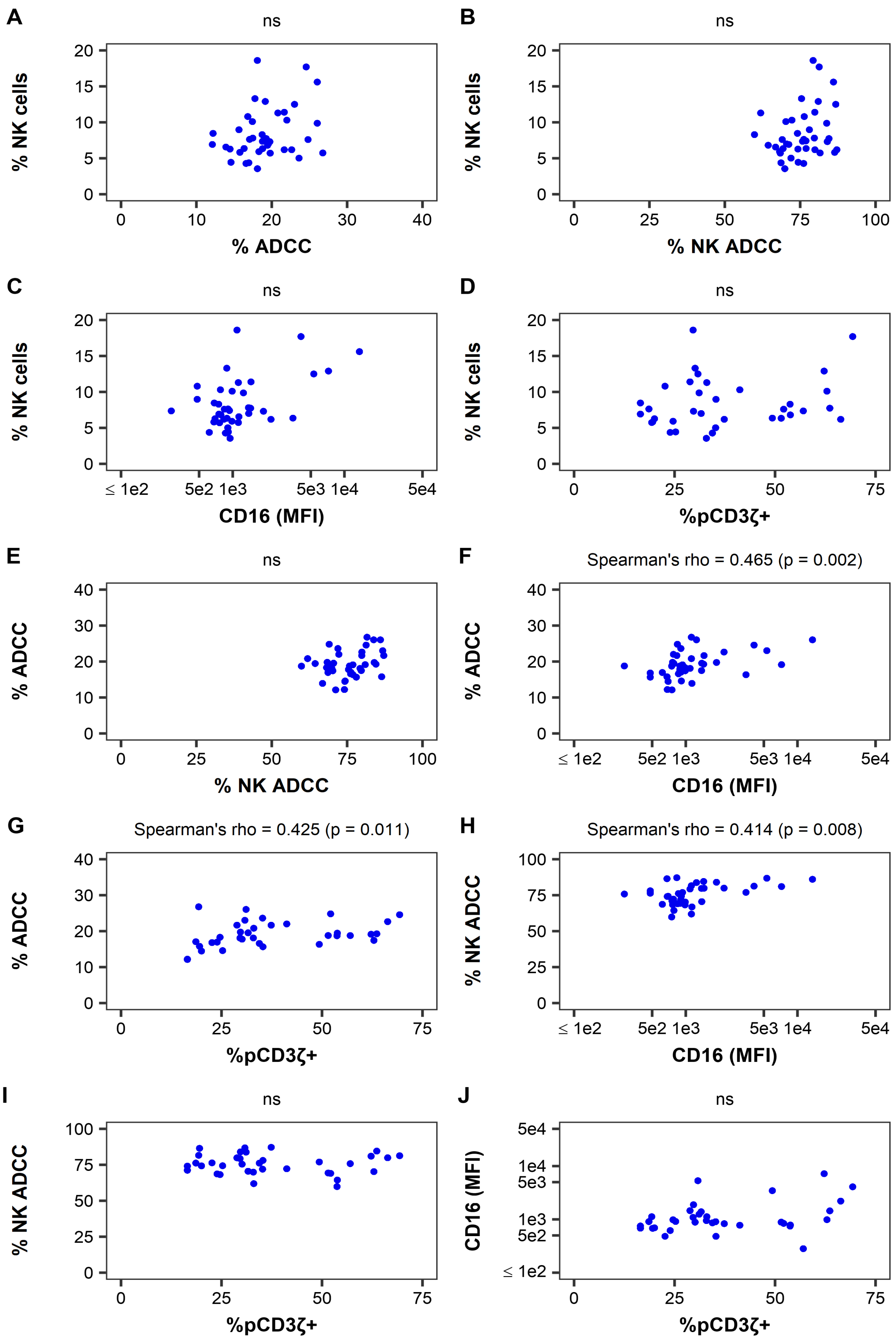

Figure S2. NK cell functions are not strongly correlated in humans. Correlation between % NK cells and ADCC (**A**), % NK cells and % NK ADCC (**B**), % NK cells and CD16 MFI (**C**) % NK cells and %pCD3ζ+ (**D**), ADCC and % NK ADCC (**E**), ADCC and CD16 MFI (**F**), ADCC and %pCD3ζ+ (**G**), % NK ADCC and CD16 MFI (**H**), % NK ADCC and %pCD3ζ+ (**I**), and CD16 MFI and %pCD3ζ+ (**J**). Spearman correlation coefficients shown only for $P < 0.05$. N=40 except in **D**, **G**, and **I – J** (N=35).

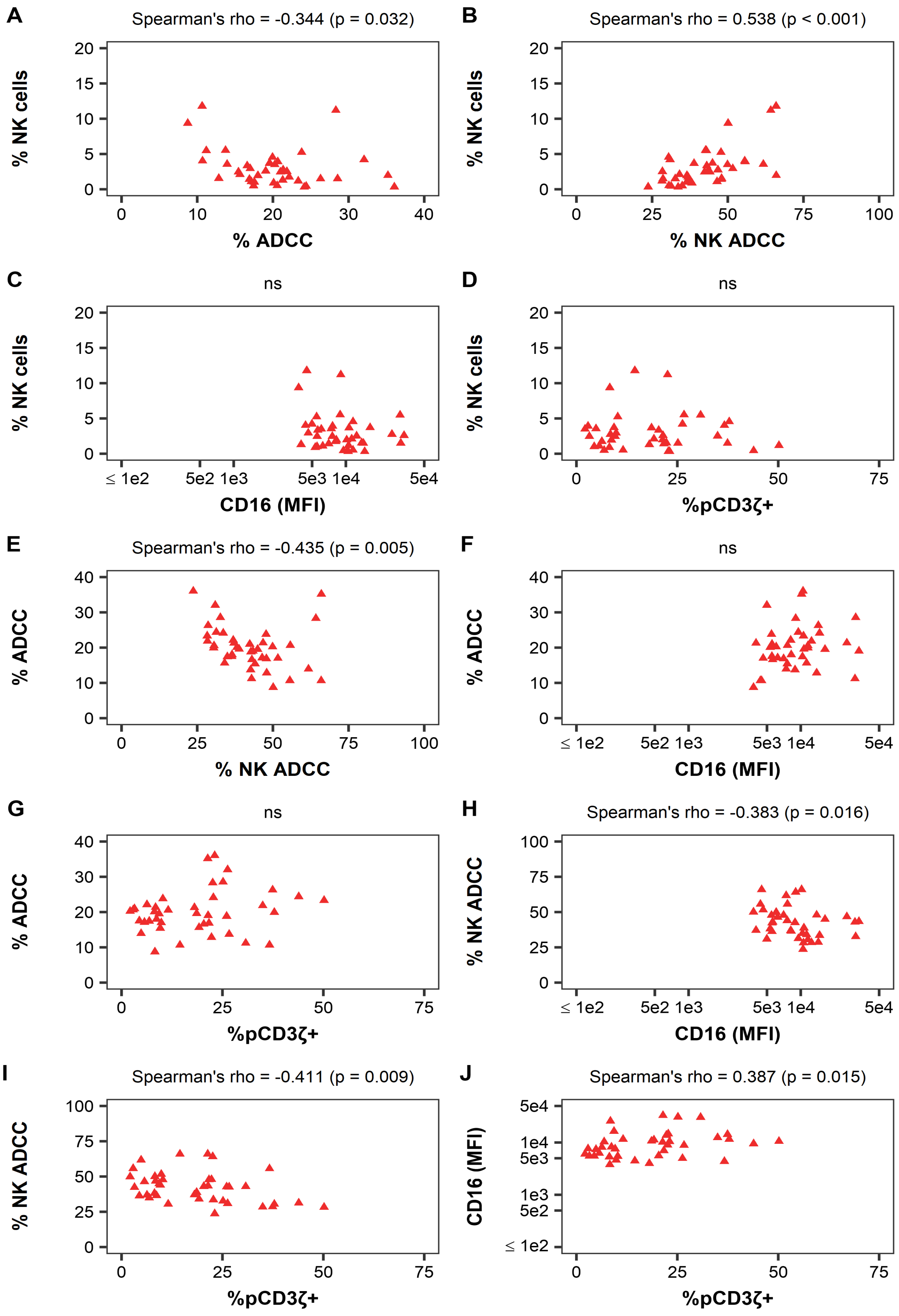

Figure S3. NK cell functions are not strongly correlated in RMs. Correlation between % NK cells and ADCC (**A**), % NK cells and % NK ADCC (**B**), % NK cells and CD16 MFI (**C**) % NK cells and %pCD3ζ+ (**D**), ADCC and % NK ADCC (**E**), ADCC and CD16 MFI (**F**), ADCC and %pCD3ζ+ (**G**), % NK ADCC and CD16 MFI (**H**), % NK ADCC and %pCD3ζ+ (**I**), and CD16 MFI and %pCD3ζ+ (**J**). Spearman correlation coefficients shown only for $P < 0.05$. N=39 in **A – D, F, H,** and **J** and N=40 in **E, G** and **I**.

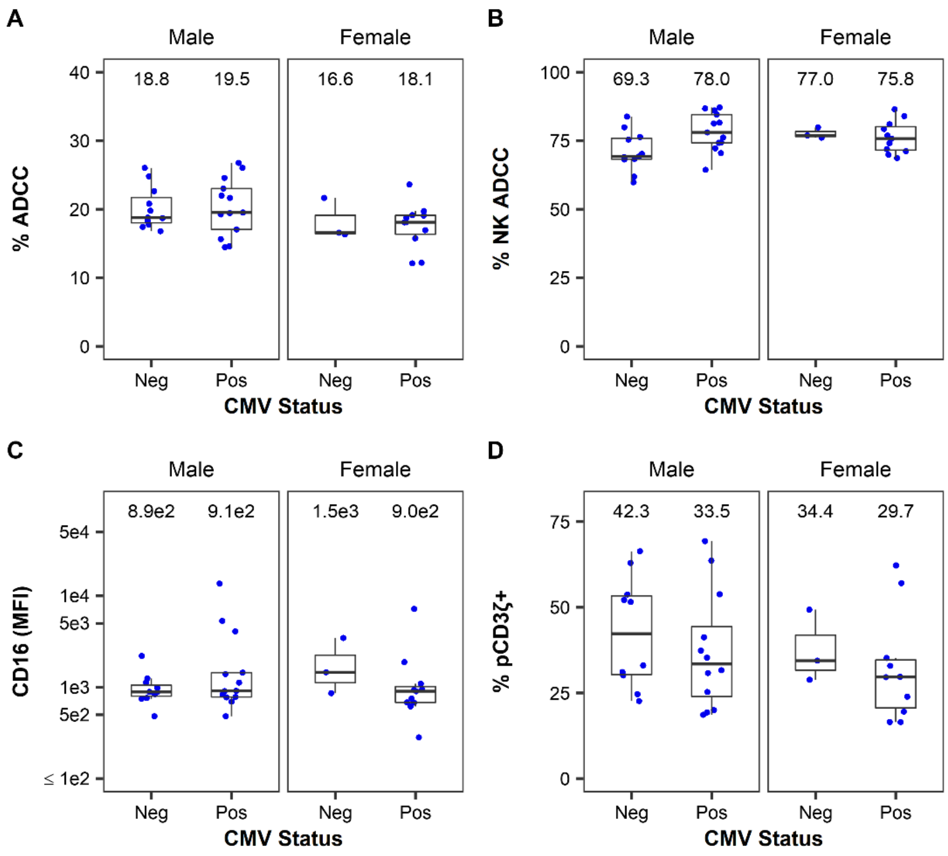

Figure S4. NK cell functions by gender and CMV status in humans. Distribution of ADCC (**A**), % NK-mediated ADCC (**B**), % NK cells (**C**), CD16 MFI (**D**), and %pCD3ζ+ (**E**) by gender and CMV status. Boxplots extend from 25th percentile to 75th percentile with a horizontal line at the median. Whiskers extend from the largest value within 25th percentile + 1.5×IQR to the smallest value within 75th percentile - 1.5×IQR, where the interquartile range (IQR) equals 75th percentile - 25th percentile. Data from each individual shown as a point. Medians are shown above the data. N=38 in **A – C**, N=35 in **D**.

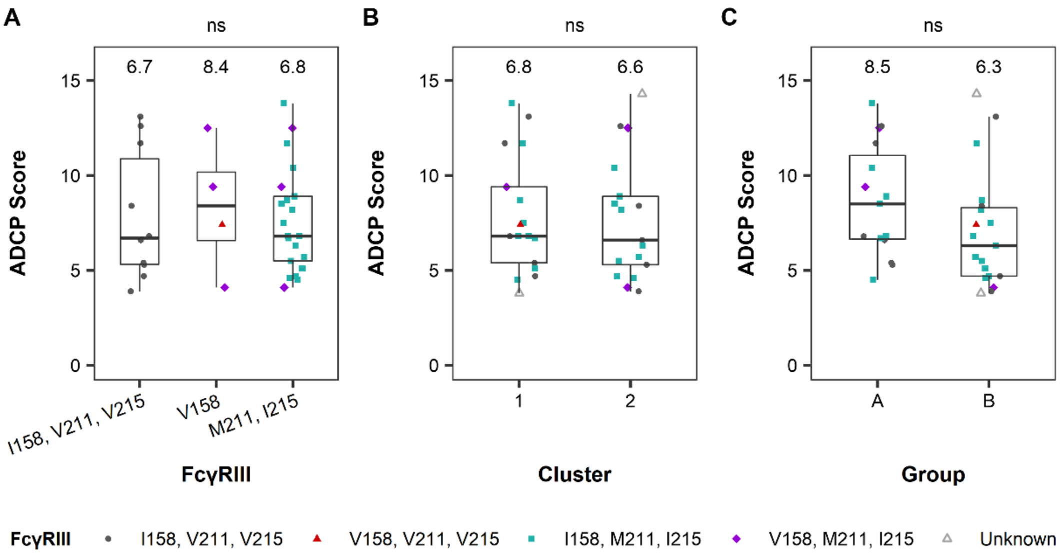

Figure S5. RM ADCP is not significantly associated with FcγRIII polymorphisms, hierarchical clusters, or covariate constrained randomization. Distribution of ADCP in RMs by polymorphisms (**A**), hierarchical clusters based on NK cell functions (**B**), and covariate constrained randomization groups based on NK cell functions (**C**). Hierarchical clusters and covariate constrained randomization groups were both based on the FcR-related NK cell functional measurements such as ADCC magnitude, % NK-mediated ADCC, CD16 expression, and %pCD3ζ+. Boxplots extend from 25th percentile to 75th percentile with a horizontal line at the median. Whiskers extend from the largest value within 25th percentile + 1.5×IQR to the smallest value within 75th percentile - 1.5×IQR, where the interquartile range (IQR) equals 75th percentile - 25th percentile. Data from each individual animal shown as a point, with color and shape by FcγRIII polymorphisms. In (**A**), RMs with V158, M211, and I215 mutations are represented twice. Medians are shown above the data. N = 32 RM in (**A**), N=34 RMs in (**B, C**). No statistically significant differences in FcR-related NK cell functions were observed based on FcγRIII polymorphisms, clusters, or randomization groups (all $P > 0.05$ by Kruskal-Wallis test (**A**) or two-sided Mann-Whitney U test (**B, C**).

Table S1. Human cohort demographics at time of blood draw. Gender, age, CMV status, weight, and FcγRIIIa genotype for human cohort.

Characteristics/Genotype	F/F N=17 (42.5%)	F/V N=18 (45%)	V/V N=5 (12.5%)
Gender			
Male	11 (27.5)	12 (30)	1 (2.5)
Female	6 (15)	6 (15)	4 (10)
Age, mean	35 (19 - 56)		
Race			
Caucasian	15 (37.5)	14 (40)	5 (12.5)
Black	2 (5)	2 (5)	0
Hispanic	0	2 (5)	0
Weight, (lbs)	197.3 (105 – 300)<br>2 Unknown	170 (125 – 224)<br>5 Unknown	174.7 (123 – 240)<br>2 Unknown
CMV status			
Pos	9 (22.5)	12 (30)	3 (7.5)
Neg	7 (17.5)	5 (12.5)	2 (5)
Intermediate	1 (2.5)	1 (2.5)	0

Table S2. Rhesus macaque cohort demographics at time of blood draw. Age, weight, and FcγRIII polymorphisms for RM cohort. All animals were CMV+ males.

NHPID	Age (years)	Weight (kg)	I158V	V211M	V215I
**JI14**	8.94	9.65	I/I	V/M	V/I
**KA56**	7.23	8.35	I/I	V/M	V/I
**KD46**	7.20	9.90	I/I	M/M	I/I
**KD71**	7.19	8.50	Unknown	Unknown	Unknown
**KE06**	7.18	8.50	I/I	V/M	V/I
**KE41**	7.17	10.00	I/I	V/V	V/V
**KJ31**	6.92	8.95	I/I	V/V	V/V
**KJ87**	6.31	8.80	I/I	V/M	V/I
**KL94**	6.24	9.70	I/I	V/M	V/I
**KM37**	6.23	9.40	I/I	V/V	V/V
**KM71**	6.22	9.50	Unknown	Unknown	Unknown
**KP85**	6.17	9.00	I/I	V/V	V/V
**KP93**	6.17	9.50	I/I	M/M	I/I
**KT15**	6.10	9.10	I/I	V/M	V/I
**LA51**	5.30	8.80	Unknown	Unknown	Unknown
**LA71**	5.26	9.70	I/I	M/M	I/I
**LA72**	5.26	8.20	I/I	V/V	V/V
**LA82**	5.26	11.60	I/I	V/M	V/I
**LB08**	5.25	9.65	I/I	V/M	V/I
**LB15**	5.24	10.40	I/I	V/V	V/V
**LB31**	5.24	7.88	I/V	V/M	V/I
**LB62**	5.23	9.05	I/V	V/V	V/V
**LC38**	5.20	9.25	I/I	M/M	I/I
**LC71**	5.19	8.95	I/V	V/M	V/I
**LD26**	5.18	9.15	I/V	V/M	V/I
**LD36**	5.18	8.80	Unknown	Unknown	Unknown
**LD37**	5.17	9.35	I/I	V/V	V/V
**LD44**	5.17	8.95	I/I	V/V	V/V
**LD54**	5.16	10.10	Unknown	Unknown	Unknown
**LD83**	5.14	8.25	I/I	V/M	V/I
**LE42**	5.11	9.20	I/I	V/V	V/V
**LE43**	5.11	10.40	I/I	V/V	V/V
**LE83**	5.08	9.60	I/I	M/M	I/I
**LF69**	4.84	9.55	I/I	V/M	V/I
**LH36**	4.21	7.95	I/I	V/M	V/I
**LH90**	5.22	9.60	I/I	M/M	I/I
**LI18**	4.16	8.30	I/I	M/M	I/I
**LI22**	4.16	8.90	I/I	V/V	V/V
**LI34**	4.15	8.00	Unknown	Unknown	Unknown
**LI54**	4.11	9.50	I/I	V/M	V/I
